# When to Best Assess Breathlessness Abnormality During Incremental Cardiopulmonary Cycle Exercise Testing

**DOI:** 10.1016/j.chest.2025.08.024

**Published:** 2025-10-09

**Authors:** Magnus Ekström, Pei Zhi Li, Jean Bourbeau, Wan C. Tan, Dennis Jensen, CanCOLD Collaborative Research Group

**Affiliations:** a Faculty of Medicine, Department of Clinical Sciences Lund, Respiratory Medicine, Allergology and Palliative Medicine, Lund University, Lund, Sweden; b Montreal Chest Institute, McGill University Health Center Research Institute, McGill University, Montréal, QC, Canada; c Translational Research in Respiratory Diseases Program and Respiratory Epidemiology and Clinical Research Unit, Research Institute of the McGill University Health Centre, Montréal, QC, Canada; d Department of Medicine, University of British Columbia Centre for Heart Lung Innovation, Vancouver, BC, Canada; e Clinical Exercise and Respiratory Physiology Laboratory, Department of Kinesiology and Physical Education, Faculty of Education, McGill University, Montréal, QC, Canada

**Keywords:** assessment, dyspnea, exercise capacity, measurement, severity

## Abstract

**Background:**

Breathlessness on exertion is a common, distressing, and limiting symptom that can be quantified on incremental cardiopulmonary exercise testing (CPET) using normative reference equations.

**Research Question:**

Is the breathlessness abnormality best uncovered and assessed at symptom limitation (peak exercise) compared with submaximal exercise intensities?

**Study Design and Methods:**

This was an analysis of people ≥ 40 years of age undergoing symptom-limited incremental cycle CPET in the Canadian Cohort Obstructive Lung Disease (CanCOLD) study. Each Borg 0-10 category ratio scale breathlessness intensity rating during CPET was converted to its probability of being normal, in relation to power output, rate of oxygen uptake, and minute ventilation using normative reference equations. Abnormally high exertional breathlessness (abnormal breathlessness) was defined as a probability of being normal < 0.05.

**Results:**

Of 1,161 participants (42% female), abnormally high breathlessness was present in 22%, 23%, and 16% in relation to rate of oxygen uptake and minute ventilation at peak exercise. Among those with abnormal breathlessness at peak exercise, 55% to 60% had normal breathlessness across all submaximal exercise intensities. Among those with normal breathlessness at peak exercise, 93% to 97% were normal across all serial breathlessness ratings throughout the CPET (interclass correlation coefficients, 0.93-0.95). Findings were similar in people with or without chronic airflow limitation, and in people who did or did not reach maximal exertion at the end (symptom limitation) of the CPET.

**Interpretation:**

The results of this study suggest that abnormal breathlessness is uncovered and should be assessed at peak exercise during symptom-limited incremental CPET. These findings inform symptom assessment in research and clinical practice.

FOR EDITORIAL COMMENT, SEE PAGE 318

**Take-Home Points**

**Research Question:** Is the abnormality (severity) of exertional breathlessness best uncovered and assessed at the symptom-limited peak compared with at submaximal exercise intensities using incremental cycle cardiopulmonary exercise testing?

**Results:** In 1,161 people (42% female), those with abnormally high exertional breathlessness at peak exercise, 55% to 60% had normal breathlessness across all submaximal exercise intensities, whereas almost all (93%-97%) of those normal at peak were normal throughout all lower exercise intensities.

**Interpretation:** Our results suggest that abnormal breathlessness is uncovered and should be assessed at peak exercise during symptom-limited incremental cardiopulmonary exercise testing, which informs symptom assessment in research and clinical practice.

Breathlessness on exertion^1^^,^^2^ is a main cause of distress and physical limitation in people living with cardiopulmonary diseases.^3^^,^^4^ Because people become less physically active to avoid the symptom, worsening breathlessness may be underdetected (hidden breathlessness), underreported, and undertreated for years and decades.^5^^,^^6^ However, data are emerging that the symptom can be unhidden using standardized exercise testing.^7^, ^8^, ^9^

Cardiopulmonary exercise testing (CPET) is the gold standard method for assessing change in exertional breathlessness and treatment effects,^10^, ^11^, ^12^ for assessing underlying physiological mechanisms,^12^ and for evaluating the symptom’s level of severity (abnormality) compared with matched healthy references.^13^, ^14^, ^15^ Normative reference equations were recently published to predict the normal breathlessness intensity response for a person at any given absolute or relative power output (W), rate of oxygen uptake ($\dot{V}$o_2_), or minute ventilation ($\dot{V}$e) throughout incremental symptom-limited cycle CPET.^14^ These methods adhere to the fundamental principle of psychophysics that valid symptom assessment needs to account for the level of symptom stimulus.^14^ Using the equations, each breathlessness intensity rating (on the Borg 0-10 category ratio scale breathlessness intensity rating [Borg CR10] scale) can be translated into its probability of normality (P_norm_), that is, the probability of observing that Borg CR10 rating among matched healthy references at that level of exertion (W and $\dot{V}$o_2_) or $\dot{V}$e,^16^ ranging from 0 (most abnormal) to 1 (most normal).^13^ Abnormally high exertional breathlessness (hereafter called abnormal exertional breathlessness) has been defined as a rating with a P_norm_ < .05 (< 5% probability among healthy references).^14^

Abnormal exertional breathlessness at peak exercise during incremental CPET has strong construct validity in people with chronic airflow limitation (CAL),^17^ can uncover exertional breathlessness missed (or underestimated) by currently used questionnaires (specifically the modified Medical Research Council [mMRC] Dyspnea Scale and COPD Assessment Test [CAT]) in people with COPD,^9^ and strongly predicts mortality even after accounting for exercise capacity.^18^

However, it is unknown at what point during the CPET that the abnormality of breathlessness is best categorized. Previous studies evaluated the symptom abnormality (P_norm_ and presence of abnormal exertional breathlessness) using the breathlessness rating at the symptom-limited peak, based on the assumption that pathophysiological abnormalities are more likely to be uncovered and/or magnified by the higher physiological stress at the end of the incremental CPET. To our knowledge, no study has evaluated how breathlessness abnormality changes from rest to the symptom-limited peak of an incremental CPET. We hypothesized the following: (1) categorizing breathlessness abnormality at peak exercise is more sensitive than using breathlessness ratings at submaximal exercise intensities, identifying a greater number of individuals with abnormal exertional breathlessness; and (2) individuals with normal breathlessness at peak exercise are also likely to show normal ratings at submaximal exercise intensities.

The aim of this study was to compare grading of breathlessness abnormality using normative reference equations in relation to W, $\dot{V}$o_2_, and $\dot{V}$e, at peak exercise compared with at any submaximal intensity during incremental cycle CPET. We also aimed to compare findings between people with or without CAL, and between people who met or did not meet criteria for reaching maximal exertion.

## Study Design and Methods

### Study Design and Population

This was an analysis of the prospective, population-based study, Canadian Cohort Obstructive Lung Disease (CanCOLD) (ClinicalTrials.gov Identifier No.: NCT00920348).^19^ Participants were noninstitutionalized male or female adults ≥ 40 years of age originally identified with random telephone digit dialing across 9 communities in Canada.^19^

All participants provided written informed consent before completing study assessments. The study was approved by the respective university and institutional ethical review boards: McGill University Health Centre Research Ethics Board, 09-025-BMB-t (Montreal); University of British Columbia/Providence Health Care Research Ethics Board, P05-006 (Vancouver); University Health Network Research Ethics Board, 06-0421-B (Toronto); Capital Health Research Ethics Board, CDHA-RS/2007-255 (Halifax); Conjoint Health Research Ethics Board, ID21258 (Calgary); Department of Medicine-1240-09 (Kingston); 2009519-01H (Ottawa); Bio-Research Ethics Board 09-162 (Saskatoon); Comité d'éthique de la recherche 20459 (Quebec City). This CanCOLD substudy is reported in accordance with the Strengthening the Reporting of Observational studies in Epidemiology statement.^20^ All data in this analysis pertain to the CanCOLD baseline visit.

**Figure 1:**
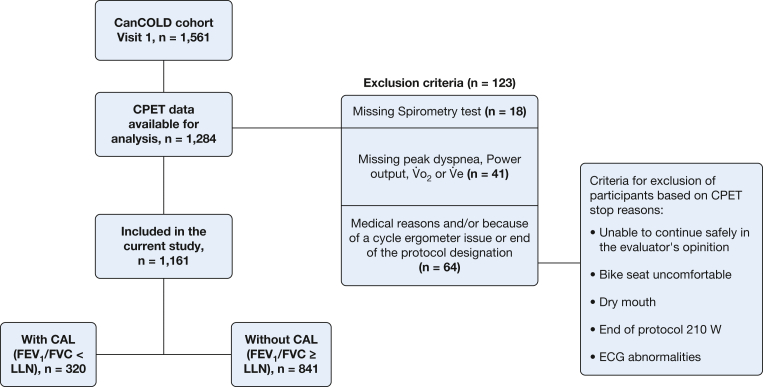
Participant flowchart. CAL = chronic airflow limitation; CanCOLD = Canadian Cohort Obstructive Lung Disease; CPET = cardiopulmonary exercise testing; LLN = lower limit of normal; $\dot{V}$e = minute ventilation; $\dot{V}$o_2_ = rate of oxygen uptake.

Eligibility criteria for this analysis were available data on spirometry; CPET data on breathlessness Borg CR10 rating, W, $\dot{V}$o_2_, and $\dot{V}$e at peak exercise; and no medical events or technical issues during the CPET (Fig 1).

### Assessments and Procedures

Participants self-reported sociodemographics and health information (eg, smoking history, presence of physician-diagnosed health conditions) via structured interview with a trained researcher. Health status was assessed using the CAT,^21^ and the impact of breathlessness on daily life activity was assessed using the mMRC.^22^ Postbronchodilator (200 μg albuterol) spirometry, diffusing capacity of the lungs for carbon monoxide, and plethysmographic lung volumes were assessed using automated equipment in accordance with published standards.^19^^,^^23^^,^^24^ Predicted lung function values were calculated using Global Lung Function Initiative references.^25^, ^26^, ^27^ CAL was defined as a postbronchodilator FEV_1_/FVC ratio < the lower limit of normal.

#### Cardiopulmonary Exercise Testing

CPET was performed in accordance with recognized guidelines^28^ on an electronically braked cycle ergometer using a computerized CPET system. The CPET protocol was standardized across CanCOLD sites and included a steady-state preexercise baseline period of 3 to 10 minutes, followed by 1 minute of unloaded pedaling, and then a 10-W/min increase in W (starting at 10 W) until symptom limitation.^29^

Gas exchange and breathing pattern parameters were collected breath-by-breath with participants breathing through a mouthpiece and flow transducer while wearing a nose clip. Heart rate and rhythm were assessed continuously by 12-lead electrocardiogram, and peripheral oxyhemoglobin saturation was monitored by finger pulse oximetry. At rest, every 2 minutes during exercise, and at peak exercise, participants performed maximal voluntary inspiratory capacity maneuvers,^30^ and rated the intensity of their perceived breathlessness and leg discomfort using the Borg CR10 scale.^31^ Before CPET, breathlessness was defined for each participant as breathing discomfort, and leg discomfort was defined as the level of discomfort experienced during pedaling; and participants were familiarized with the Borg CR10 scale such that 0 represented no breathing (leg) discomfort and 10 represented the most severe breathing (leg) discomfort that you have ever experienced or can imagine experiencing. Peak W was taken as the highest W a participant was able to sustain for ≥ 30 seconds, whereas peak $\dot{V}$o_2_ and peak $\dot{V}$e were taken as the average of the last 30 seconds of loaded pedaling.

#### Physiological Responses to CPET

Physiological variables evaluated during CPET included the following: exercise capacity as peak W and peak $\dot{V}$o_2_; dynamic hyperinflation as change in inspiratory capacity from preexercise baseline to peak exercise indexed to peak $\dot{V}$e (with lower values indicating greater dynamic hyperinflation); ventilatory inefficiency as the nadir of the ventilatory equivalent for CO_2_, identified as the lowest 30-second average data point observed during CPET; and critical inspiratory constraint as the end-inspiratory lung volume-to-total lung capacity ratio indexed to peak $\dot{V}$e. Unless indicated otherwise, all physiological variables used in the analyses were values obtained at the symptom limited peak of exercise. Predicted values for peak W, peak $\dot{V}$o_2_, peak $\dot{V}$e, and nadir ventilatory inefficiency as the nadir of the ventilatory equivalent for CO_2_ were calculated using CanCOLD references.^29^

#### Abnormal Exertional Breathlessness

For each breathlessness rating from rest to peak exercise, abnormally high exertional breathlessness was defined as a Borg CR10 intensity rating > upper limit of normal (ULN), in relation to the contemporaneous W, $\dot{V}$o_2_, and $\dot{V}$e separately, using published normative reference equations.^14^ The reference equations were developed in ostensibly healthy individuals in the CanCOLD cohort as detailed elsewhere.^14^

### Statistical Analyses

Characteristics were summarized using mean ± SD and median with range or interquartile range for continuous variables, as appropriate. Categorical variables were expressed as frequencies and percentages. Participants were categorized based on the breathlessness intensity response at the symptom limited peak of exercise into those with abnormally high exertional breathlessness (rating > ULN) or normal breathlessness (rating ≤ ULN). We evaluated all breathlessness ratings throughout the CPET from rest to peak exercise for each individual by plotting the proportion with abnormally high exertional breathlessness and the P_norm_ at each stage of the CPET by each physiological variable (W, $\dot{V}$o_2_, and $\dot{V}$e) in relation to the individual’s achieved peak value (% peak) in 10% intervals. Because comparisons were between breathlessness ratings from rest to peak within each individual, all analyses are controlled by design for interindividual differences.

To evaluate the secondary aims, analyses were performed for all participants and separately in people with or without (1) CAL, and (2) a maximal test, defined as having a respiratory exchange rate of ≥ 1.0 or heart rate at peak exercise no less than 10 beats/min below the age-predicted reference value of 220 − age.^29^ Consistency of exertional breathlessness abnormality across all symptom ratings throughout the CPET (ie, from rest to peak) was evaluated, for participants with at least 3 breathlessness ratings, using the interclass correlation coefficient (ICC) from random intercept GLIMMIX procedure with logit link function and variance components structure, where ICC = covariance parameter estimate/(covariance parameter estimates + 3.29), with 3.29 approximating the variance of a standard logistic function; 95% CI for ICC was estimated via Fisher *z* transformation. The ICC ranges from 0 (no consistency) to 1.0 (perfect consistency), with values < 0.5 considered as poor, 0.50 to 0.75 as moderate, 0.76 to 0.90 as good, and > 0.90 as excellent consistency.^32^ Statistical analyses were conducted using the SAS version 9.4 software (TS1M5) (SAS Institute Inc).

## Results

A total of 1,161 people (42% female) were included in the analyses (Fig 1). Participants had a mean age of 66 ± 9.8 years (range, 40-91), and 320 (28%) had CAL (Table 1). Abnormal exertional breathlessness at peak exercise was present in 22% relative to W, 23% relative to $\dot{V}$o_2_, and 16% relative to $\dot{V}$e. The median number of breathlessness ratings throughout the CPET, from rest to peak exercise, was 6 (interquartile range, 5-8) per participant (Table 1).

**Table 1:** Participant Characteristics

Characteristic	All Participants (N = 1,161)	With CAL (n = 320; 28%)	Without CAL (n = 841; 72%)
Age, y	66.3 [9.8] (40.0-91.0)	64.4 [10.2] (40.0-89.0)	67.1 [9.6] (42.0-91.0)
Women	490 (42.2)	139 (43.4)	351 (41.7)
BMI, kg/m^2^	27.4 (4.9)	27.2 (4.9)	27.5 (4.9)
Obesity (BMI ≥ 30 kg/m^2^)	292 (25.2)	84 (26.3)	208 (24.7)
Cigarette smoking status			
Has never smoked	463 (39.9)	90 (28.1)	373 (44.4)
Formerly smoked	534 (46.0)	160 (50.0)	374 (44.5)
Currently smokes	164 (14.1)	70 (21.9)	94 (11.2)
Cigarette pack-years	17.0 [22.1]	25.8 [24.8]	13.6 [19.9]
Hypertension	391 (33.7)	100 (31.3)	291 (34.6)
Any cardiovascular diseases (excluding hypertension)	322 (27.7)	80 (25.0)	242 (28.8)
Physician-diagnosed COPD	205 (17.7)	122 (38.1)	83 (9.9)
Physician-diagnosed asthma	280 (24.1)	136 (42.5)	144 (17.1)
Any current respiratory medication(s) use	276 (23.8)	161 (50.3)	115 (13.7)
mMRC breathlessness rating			
0	695 (62.4)	144 (46.5)	551 (68.6)
1	359 (32.3)	133 (42.9)	226 (28.1)
≥ 2	59 (5.3)	33 (10.6)	26 (3.2)
CAT total score	6.7 (5.6)	9.0 (6.9)	5.8 (4.8)
Lung function at rest			
FEV_1_, % predicted	91.8 (19.4)	75.2 (17.5)	98.1 (16.1)
GOLD stage			
GOLD 1 (FEV_1_/FVC < LLN and FEV_1_ ≥ 80% predicted)	130 (11.1)	130 (40.6)	0 (0)
GOLD 2 (FEV_1_/FVC < LLN and FEV_1_ ≥ 50% and < 80% predicted)	161 (13.8)	161 (50.3)	0 (0)
GOLD 3 (FEV_1_/FVC < LLN and FEV_1_ ≥ 30% and < 50% predicted)	28 (2.4)	28 (8.8)	0 (0)
GOLD 4 (FEV_1_/FVC < LLN and FEV_1_ < 30% predicted)	1 (0.1)	1 (0.3)	0 (0)
FVC, % predicted	102.1 (17.1)	101.9 (19.2)	102.2 (16.3)
FEV_1_/FVC, %	69.5 (10.2)	57.1 (8.0)	74.2 (6.2)
TLC, % predicted	105.7 (15.1)	110.0 (15.2)	104.0 (14.8)
IC, % predicted	98.5 (23.5)	94.9 (22.7)	99.9 (23.7)
Dlco, % predicted	93.4 (22.1)	87.8 (23.3)	95.6 (21.2)
CPET parameters at symptom-limited peak exercise			
Physiological responses			
Power output, % predicted	88.4 [25.0]	80.9 [24.6]	91.2 [24.5]
HR, % predicted	93.3 [15.5]	90.5 [15.2]	94.3 [15.4]
Respiratory exchange ratio	1.10 [0.11]	1.09 [0.12]	1.10 [0.10]
$\dot{V}$o_2_, L/min	1.7 [0.6]	1.6 [0.6]	1.7 [0.6]
$\dot{V}$o_2_, % predicted	88.7 [23.6]	82.4 [22.6]	91.0 [23.6]
$\dot{V}$co_2_, L/min	1.8 [0.7]	1.7 [0.7]	1.9 [0.7]
$\dot{V}$e, % predicted	90.4 [25.9]	83.0 [24.4]	93.2 [25.9]
Nadir $\dot{V}$e/$\dot{V}$co_2_	31.0 [6.0]	32.1 [6.8]	30.6 [5.6]
Δ IC/$\dot{V}$e	−0.002 [0.008]	−0.004 [0.009]	−0.001 [0.007]
EILV%TLC/$\dot{V}$e	1.7 [0.7]	1.8 [0.7]	1.6 [0.7]
Breathlessness responses			
Breathlessness (Borg CR10), median (Q1-Q3)	5.0 (3.0-7.0)	5.0 (4.0-7.0)	5.0 (3.0-7.0)
P_norm_ by W equation	0.30 (0.29)	0.26 (0.28)	0.32 (0.30)
P_norm_ by $\dot{V}$o_2_ equation	0.31 (0.30)	0.27 (0.28)	0.32 (0.31)
P_norm_ by $\dot{V}$e equation	0.37 (0.31)	0.34 (0.31)	0.39 (0.32)
Breathlessness abnormality (> ULN) by W equation	256 (22.0)	85 (26.6)	171 (20.3)
Breathlessness abnormality (> ULN) by $\dot{V}$o_2_ equation	264 (22.7)	79 (24.7)	185 (22.0)
Breathlessness abnormality (> ULN) by $\dot{V}$e equation	184 (15.8)	59 (18.4)	125 (14.9)
No. of breathlessness ratings throughout the CPET, median (Q1-Q3) (minimum-maximum]	6.0 (5.0-8.0) (2.0-14.0)	6.0 (5.0-7.5) (2.0-14.0)	7.0 (5.0-8.0) (2.0-13.0)

Data are presented as mean [SD] (minimum-maximum), mean [SD], No. (%), or as otherwise indicated. Borg CR10 = Borg 0-10 category ratio scale breathlessness intensity rating; CAL = chronic airflow limitation; CAT, COPD Assessment Test; CPET = cardiopulmonary exercise testing; Dlco = diffusion lung capacity for carbon monoxide; EILV = end inspiratory lung volume; GOLD = Global Initiative for Obstructive Lung Disease; HR = heart rate; IC = inspiratory capacity; LLN = lower limit of normal; mMRC = modified Medical Research Council dyspnea score; P_norm_ = probability of normality; Q = quartile; TLC = total lung capacity; $\dot{V}$co_2_ = rate of carbon dioxide production; $\dot{V}$e = minute ventilation; $\dot{V}$o_2_ = rate of oxygen uptake; W = power output.

**Figure 2:**
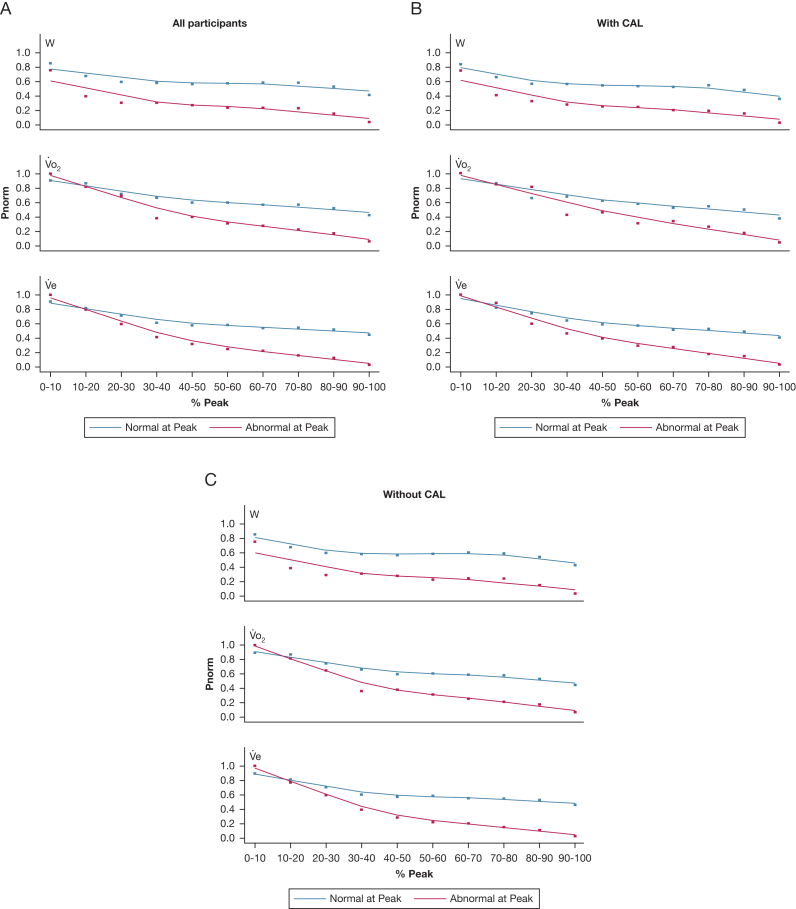
A-C, P_norm_ Borg 0-10 category ratio scale breathlessness intensity ratings throughout the symptom-limited incremental cardiopulmonary cycle exercise test (CPET) compared between those with abnormal vs normal breathlessness at peak exercise: (A) all participants, (B) people with CAL, and (C) people without CAL. The x axis variable is the W, $\dot{V}$o_2_, or $\dot{V}$e, expressed as a percentage of the obtained peak value for each individual, to enable comparisons of the trajectories from the start to end of CPET. A lower P_norm_ (on y axis) reflects more abnormal (severe) exertional breathlessness. CAL = chronic airflow limitation; P_norm_ = probability of normality; $\dot{V}$e = minute ventilation; $\dot{V}$o_2_ = rate of oxygen uptake; W = power output.

**Figure 3:**
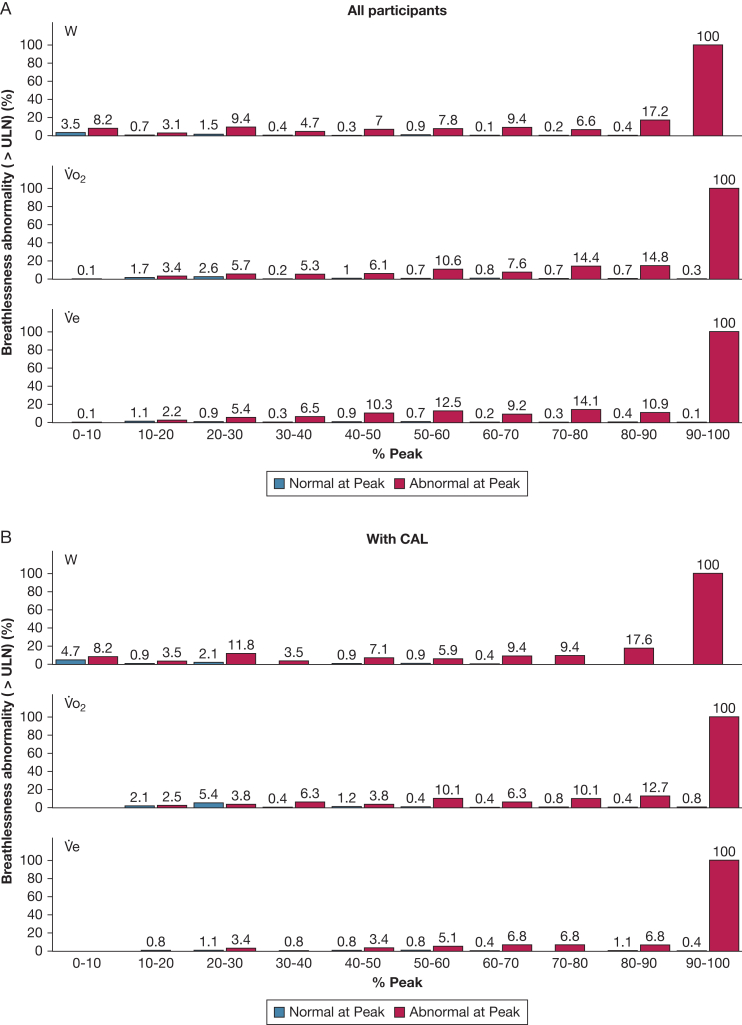

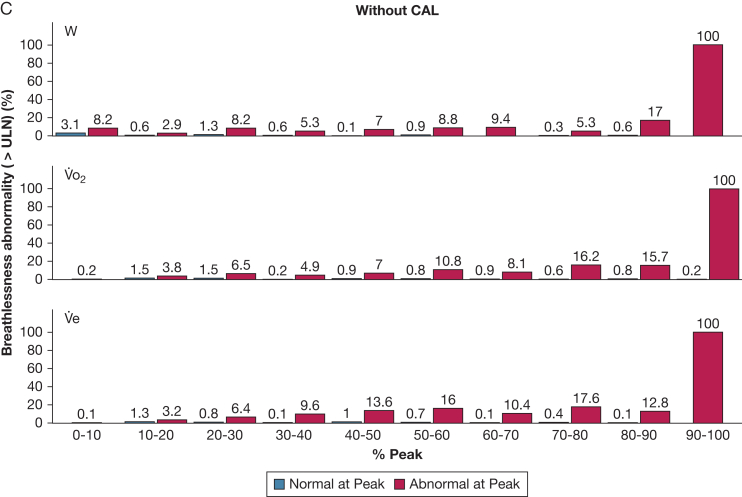
A-C, Percentage of people with abnormally high exertional breathlessness at different stages of the symptom-limited incremental cycle cardiopulmonary exercise test (CPET) compared between those with abnormal vs normal breathlessness at peak exercise: (A) all participants, (B) people with CAL, and (C) people without CAL. The x axis variable is W, $\dot{V}$o_2_, or $\dot{V}$e, expressed as a percentage of the obtained peak value for each individual, to enable comparisons of the trajectories from the start to end of CPET. Abnormal exertional breathlessness was defined as a Borg 0-10 category ratio scale breathlessness intensity rating above the predicted ULN, using normative reference equations.^14^ CAL = chronic airflow limitation; ULN = upper limit of normal; $\dot{V}$e = minute ventilation; $\dot{V}$o_2_ = rate of oxygen uptake; W = power output.

Breathlessness abnormality (P_norm_ and the proportion of ratings being abnormal) from rest to peak exercise is compared between people who had abnormal or normal breathlessness at peak exercise in Figures 2 and 3. Among those with abnormal breathlessness at peak exercise, the breathlessness became, on average, progressively more abnormal (decreasing P_norm_) with increasing W, $\dot{V}$o_2_, and $\dot{V}$e from rest to peak exercise, and the differences compared with the normal group became progressively larger throughout the CPET (Fig 2). Most people with abnormal breathlessness at peak exercise had normal breathlessness intensity ratings at submaximal exercise intensities (Fig 3); as many as 55% to 59% of people with abnormally high breathlessness at peak exercise had breathlessness intensity ratings within normal predicted limits (≤ ULN) up to the point of symptom-limited peak exercise (Table 2).

**Table 2:** Consistency of Abnormal and Normal Exertional Breathlessness Throughout Symptom-Limited Incremental Cardiopulmonary Cycle Exercise Testing

Response Pattern	All Participants (N = 1,156)					
	W Equation		$\dot{V}$o_2_ Equation		$\dot{V}$e Equation	
	Normal at Peak	Abnormal at Peak	Normal at Peak	Abnormal at Peak	Normal at Peak	Abnormal at Peak
	(n = 902)	(n = 254)	(n = 893)	(n = 263)	(n = 973)	(n = 183)
Consistently normal	845 (93.7)	NA	834 (93.4)	NA	935 (97.1)	NA
Abnormal-normal	57 (6.3)	NA	59 (6.6)	NA	38 (3.9)	NA
All normal before peak-peak abnormal	NA	150 (59.1)	NA	148 (56.3)	NA	100 (54.6)
Abnormal-peak abnormal (consistent abnormal from the first abnormal)	NA	72 (28.4)	NA	76 (28.9)	NA	62 (33.9)
Abnormal-normal-peak abnormal (instability)	NA	32 (12.6)	NA	39 (14.8)	NA	21 (11.5)
ICC (95% CI) (all ratings)	0.93 (0.92 to 0.94)	0.11 (−0.01 to 0.23)	0.93 (0.92 to 0.94)	0.11 (−0.01 to 0.23)	0.95 (0.94 to 0.96)	0.09 (−0.05 to 0.23)

Values are for participants with at least 3 breathlessness ratings during the cardiopulmonary exercise test (excluding 5 participants with only 2 ratings). Data are presented as No. (%) or as otherwise indicated. ICC = interclass correlation coefficient; NA = not applicable; $\dot{V}$e = minute ventilation; $\dot{V}$o_2_ = rate of oxygen uptake; W = power output.

Of participants with normal breathlessness at peak exercise, 93% to 97% had breathlessness intensity ratings within normal predicted limits at all submaximal exercise intensities (Fig 3). Thus, normal breathlessness showed excellent consistency throughout the CPET with an ICC of 0.93 to 0.95 (Table 2).

Findings were similar for the equations in relation to W, $\dot{V}$o_2_, and $\dot{V}$e (Figure 2, Figure 3; Table 2), between people with or without CAL (e-Table 1; Figure 2, Figure 3), and in people who did not reach the criteria for maximal exercise test (n = 168; 14%) (e-Table 2).

## Discussion

### Main Findings

This study shows that using the last (peak) measurements has the highest performance to identify and grade the abnormality (severity) of exertional breathlessness using CPET. This finding was similar for breathlessness in relation to level of exertion (W and $\dot{V}$o_2_) and $\dot{V}$e, in people with or without CAL, and in people who met or did not meet criteria for reaching maximal exertion.

### What This Study Adds

These findings inform how we should grade abnormality of exertional breathlessness in research and clinical care. The result that breathlessness ratings at peak exercise (standardized to the individual’s W, $\dot{V}$o_2_, and $\dot{V}$e using normative reference equations^14^) was most sensitive to identify abnormally high exertional breathlessness supports the assumption that pathophysiological abnormalities are more likely to be magnified, uncovered, and/or detected during incremental exercise testing at the limits of tolerance when cardiometabolic and ventilatory demands are high. In the present study, a person whose breathlessness intensity ratings was within normal limits at peak exercise was also very likely to report breathlessness within normal limits at submaximal exercise intensities throughout CPET (94% were normal across all breathlessness intensity ratings with an ICC of 0.93). On the other hand, abnormal exertional breathlessness (rating > ULN) was uncovered for most people only at the symptom-limited peak of CPET. Indeed, of those people with abnormally high exertional breathlessness at peak exercise, 55% to 59% had breathlessness intensity ratings within normal limits at all submaximal exercise intensities, and only 28% to 34% reported abnormally high exertional breathlessness at all submaximal and peak measurements during the CPET. Importantly, abnormal exertional breathlessness at peak exercise during incremental CPET has high construct validity and prognostic value^8^^,^^9^^,^^17^^,^^18^—it is strongly linked to worse physiological responses at rest (eg, more airflow limitation, lower diffusing capacity of the lungs for carbon monoxide) and during CPET (eg, low peak $\dot{V}$o_2_ and greater critical inspiratory constraint, dynamic hyperinflation and exercise ventilatory inefficiency) and to worse self-reported physical activity, symptoms, and health status in daily life (eg, higher mMRC and CAT scores) in people with CAL.^17^ Exertional breathlessness evaluated using normative reference equations at peak exercise was also more sensitive than mMRC and CAT to identify people with COPD who were symptomatic and had more physiological impairments at CPET.^9^ Abnormally high exertional breathlessness evaluated using normative reference equations at peak exercise is also an independent predictor of all-cause, respiratory, and cardiovascular mortality.^18^

### Implications

These findings have important implications. For optimal sensitivity and validity, exertional breathlessness should be graded using intensity ratings obtained at the highest available exercise intensity during standardized exercise testing, preferably peak exercise. Normative reference equations are available to do this both for symptom-limited incremental cycle CPET^14^^,^^15^ and the 6-minute walk test.^33^ Our findings also inform the choice of test to detect and grade exertional breathlessness because the probability of uncovering abnormally high intensity ratings improves at an individual’s limit of tolerance when cardiometabolic and ventilatory demands are highest. Compared with evaluations of breathlessness at nonstandardized levels of exertion (eg, recall during daily life activities using questionnaires like the mMRC), or at submaximal exercise intensities, the sensitivity and validity of the breathlessness evaluation will be optimized when assessed using a symptom-limited, incremental CPET at peak exercise.

Another important implication of our findings is that, when determining study designs and the timing of exertional breathlessness assessments, the most critical measurement should be taken at the symptom-limited peak. An advantage is that assessment at peak allows both evaluation of exercise capacity and the (ab)normality of exertional symptoms, which may reduce the number of tests needed and the burden on individuals. Of note, the raw peak breathlessness intensity rating (eg, Borg CR10) should not be used in isolation because individuals—regardless of health status or disease severity—tend to stop exercising at similar symptom thresholds, despite reaching vastly different levels of exertion (W and $\dot{V}$o_2_) and/or $\dot{V}$e.^34^, ^35^, ^36^ Therefore, peak breathlessness intensity ratings should be standardized for the level of exertion and/or $\dot{V}$e using normative reference equations.^13^

### Strengths and Limitations

The strengths of this study include the large population-based sample of adults ≥ 40 years of age with and without CAL who underwent detailed physiological measurements at rest and during standardized CPET. A limitation is the low number of people with severe CAL, which should be evaluated in future studies. However, these findings are likely applicable to both in healthy individuals and those with varying degrees of disease severity. This is supported by the similar results observed in people with or without CAL, and aligns with the concept that the sensitivity and validity of detecting and grading pathophysiological abnormalities improve with increasing exercise intensity (and cardiometabolic and ventilatory demands) during incremental exercise testing. Breathlessness that is underreported and hidden at rest or during low levels of physical activity is likely to be revealed at the symptom-limited peak of standardized exercise testing.^7^^,^^9^^,^^37^

Future research should evaluate whether the trajectory of exertional breathlessness abnormality throughout the exercise test, despite the low rate of abnormal breathlessness at submaximal exercise intensities, may add further clinical or prognostic information in different patient populations (eg, people undergoing clinical evaluation for unexplained breathlessness). The present findings pertain to incremental cycle CPET. Symptom assessment using normative reference equations should also be evaluated for constant-load CPET and for tests using treadmill vs cycle ergometer. In addition, breathlessness is multidimensional and we recently showed that breathing sensations (rated using the Multidimensional Dyspnea Profile) at peak exercise were similar between people with normal and abnormal exertional breathlessness.^38^ That finding supports the notion that people, across health and disease, tend to exercise up to a relatively similar symptom level (breathing sensations)—and that dimensions of exertional breathlessness also need to be evaluated in relation to the person’s characteristic and the underlying stimulus.^38^ Therefore, normative reference equations should be developed for measuring breathlessness dimensions scores during standardized exercise testing.

## Interpretation

This trial showed that during incremental CPET, the severity (abnormality) of exertional breathlessness is best graded using the last (peak) assessment. This was seen similarly for breathlessness evaluated in relation to exertion (W and $\dot{V}$o_2_) or $\dot{V}$e, in people without and with CAL, and in people who met or did not meet criteria for reaching maximal exertion. These findings inform how to best measure and compare the abnormality (severity) of exertional breathlessness in research and clinical care.

## Funding/Support

The CanCOLD study (ClinicalTrials.gov Identifier No.: NCT00920348) has received support from the 10.13039/501100021743Canadian Respiratory Research Network, the Canadian Institutes of Health Research [CIHR/Rx&D Collaborative Research Program Operating Grant 93326], the Respiratory Health Research Network of the Fonds de la Recherche en Santé du Québec, the Foundation of the 10.13039/100014131McGill University Health Centre, and industry partners, including the following: 10.13039/100008207AstraZeneca Canada Ltd, Boehringer Ingelheim Canada Ltd, 10.13039/100004330GlaxoSmithKline (GSK) Canada Ltd, Novartis, Almirall, Merck, Nycomed, Pfizer Canada Ltd, and Theratechnologies. M. E. was supported by an unrestricted grant from the Swedish Research Council [Dnr: 2019-02081]. D. J. holds a Canada Research Chair, Tier II, in Clinical Exercise & Respiratory Physiology from the 10.13039/501100000024Canadian Institutes of Health Research.

## Financial/Nonfinancial Disclosures

The authors have reported to *CHEST* the following: J. B. and W. C. T. report receiving institutional funding for the CanCOLD study from Astra Zeneca Canada Ltd, Boehringer-Ingelheim Canada Ltd, GlaxoSmithKline Canada Ltd, Merck, Novartis Pharma Canada Inc, and Nycomed Canada Inc (W. C. T.), Pfizer Canada Ltd (W. C. T.), Trudell (J. B.), and Grifolds (J. B.). None declared (M. E., P. Z. L., D. J.).

## Supplementary Data

e-Online Data
