# Supplementary material for: When to Best Assess Breathlessness Abnormality During Incremental Cardiopulmonary Cycle Exercise Testing
Source: Chest. 2025 Oct 9;169(2):449–61. doi: 10.1016/j.chest.2025.08.024 (PMC12895330; doi:10.1016/j.chest.2025.08.024)
Supplement: e-Online Data [file mmc1.pdf]

SUPPLEMENTAL MATERIAL

Title: Breathlessness abnormality is best assessed at peak exercise during incremental cardiopulmonary cycle exercise testing

e-Table 1. Consistency of abnormal and normal exertional breathlessness throughout symptom-limited incremental cardiopulmonary cycle exercise testing, in people with or without chronic airflow limitation (CAL).

	**With CAL (n=318)**						**Without CAL (n=838)**					
	**W equation**		**V'O_2 equation**		**V'$_E$ equation**		**W equation**		**V'O_2 equation**		**V'$_E$ equation**	
	**Normal group at peak**	**Abnormal group at peak**	**Normal group at peak**	**Abnormal group at peak**	**Normal group at peak**	**Abnormal group at peak**	**Normal group at peak**	**Abnormal group at peak**	**Normal group at peak**	**Abnormal group at peak**	**Normal group at peak**	**Abnormal group at peak**
	n=234	n=84	n=240	n=78	n=260	n=58	n=668	n=170	n=653	n=185	n=713	n=125
Consistent Normal, n (%)	217 (92.7%)	-	216 (90.0%)	-	248 (95.4%)	-	628 (94.0%)	-	618 (94.6%)	-	687 (96.4%)	-
Abnormal-Normal, n (%)	17 (7.3%)	-	24 (10.0%)	-	12 (4.6%)	-	40 (6.0%)	-	35 (5.4%)	-	26 (3.6%)	-
All normal before peak-Peak Abnormal, n (%)	-	45 (53.6%)	-	47 (60.3%)	-	38 (65.5%)	-	105 (61.8%)	-	101 (54.6%)	-	62 (49.6%)
Abnormal-Peak Abnormal (consistent abnormal from the first abnormal), n (%)	-	25 (29.8%)	-	20 (25.6%)	-	16 (27.6%)	-	47 (27.7%)	-	56 (30.3%)	-	46 (36.8%)
Abnormal-Normal-Peak Abormal (instability), n (%)	-	14 (16.7%)	-	11 (14.1%)	-	4 (6.9%)	-	18 (10.6%)	-	28 (15.1%)	-	17 (13.6%)

e-Table 2. Consistency of abnormal and normal exertional breathlessness throughout symptom-limited incremental cardiopulmonary cycle exercise testing, in people meeting or not meeting criteria for maximal exercise test.

	**Participants with a maximal test (n=988)**		**Without maximal test (n=168)**		**P-value between the groups**	
	**Normal at peak**	**Abnormal at peak**	**Normal at peak**	**Abnormal at peak**	**Normal group at peak**	**Abnormal group at peak**
**W equation, n (%)**	**n=783**	**n=205**	**n=119**	**n=49**		
Consistent Normal, n (%)	736 (94.0)	-	109 (91.6)	-	0.317	-
Abnormal-Normal, n (%)	47 (6.0)	-	10 (8.4)	-	0.317	-
All normal before peak-Peak Abnormal, n (%)	-	125 (61.0)	-	25 (51.0)	-	0.203
Abnormal-Peak Abnormal (consistent abnormal from the first abnormal), n (%)	-	52 (25.4)	-	20 (40.8)	-	0.031*
Abnormal-Normal-Peak Abormal (instability), n (%)	-	28 (13.7)	-	4 (8.2)	-	0.349
**V’O_2 equation, n (%)**	**n=767**	**n=221**	**n=126**	**n=42**		
Consistent Normal, n (%)	718 (93.6)	-	116 (92.1)	-	0.517	-
Abnormal-Normal, n (%)	49 (6.4)	-	10 (7.9)	-	0.517	-
All normal before peak-Peak Abnormal, n (%)	-	128 (57.9)	-	20 (47.6)	-	0.217
Abnormal-Peak Abnormal (consistent abnormal from the first abnormal), n (%)	-	57 (25.8)	-	19 (45.2)	-	0.011*

Abnormal-Normal-Peak Abormal (instability), n (%)	-	36 (16.3)	-	3 (7.1)	-	0.158
**V'_E equation, n (%)**	**N=843**	**N=145**	**N=130**	**n=38**		
Consistent Normal, n (%)	811 (96.2)	-	124 (95.4)	-	0,654	-
Abnormal-Normal, n (%)	32 (3.8)	-	6 (4.6)	-	0,654	-
All normal before peak-Peak Abnormal, n (%)	-	83 (57.2)	-	17 (44.7)	-	0.168
Abnormal-Peak Abnormal (consistent abnormal from the first abnormal), n (%)	-	45 (31.0)	-	17 (44.7)	-	0.112
Abnormal-Normal-Peak Abormal (instability), n (%)	-	17 (11.7)	-	4 (10.5)	-	1.000

Participants with a maximal test (n=988) included 89.6%, 0.4% and 10.0% meeting peak RER criteria, peak HR criteria and both peak RER and peak HR critieria, respectively. The criteria are listed in Figure 1. For abbreviations see Table 1.
